# Higher baseline BMI and lower estimated median income associated with increasing BMI after endometrial cancer diagnosis

**DOI:** 10.1016/j.gore.2022.101123

**Published:** 2022-12-13

**Authors:** Shariska Petersen Harrington, Julia Balmaceda, Lori Spoozak, Andrea Jewell, Sharon Fitzgerald-Wolff

**Affiliations:** a Division of Gynecologic Oncology, Department of Obstetrics & Gynecology, University of Kansas Medical Center, Kansas City, KS, United States; b University of Kansas School of Medicine, University of Kansas Medical Center, Kansas City, KS, United States; c Department of Population Health, The University of Kansas Medical Center, Kansas City, KS, United States

**Keywords:** Endometrial cancer, Increasing BMI, Class III obesity

## Abstract

**Introduction:**

Endometrial cancer is often directly related to obesity and interventions for weight loss have mixed results. Risk factors for continued weight gain after diagnosis are not clearly defined in the literature. The objective of this study is to describe risk factors associated with increased body mass index (BMI) trajectory among endometrial cancer patients.

**Methods:**

Patients who were surgically treated for endometrial cancer at a single institution between 2010 and 2015 were identified. Demographics including age, race/ethnicity and estimated median income at diagnosis were obtained. BMI at five time points after diagnosis were calculated. BMI trajectories were estimated by latent class growth modeling using the PROC TRAJ procedure in SAS. Chi-squared tests and ANOVA were used to assess differences between trajectory groups. Statistical significance was set to a p-value < 0.05.

**Results:**

Of 695 patients included in the study, the average age at diagnosis was 62 years and over 70% of patients were obese at baseline. Patients experienced increasing, stable, or decreasing BMI over 2 years following diagnosis. Patients with younger age and lower estimated median income were most likely to be in the increasing BMI group. Among obese patients, those with Class I obesity (BMI 30 to 34.9 kg/m^2^) were most likely to experience decreasing BMI and those with Class III obesity (BMI > 40 kg/m^2^) were most likely to experience increasing BMI, p < 0.0001.

**Conclusion:**

A third of endometrial cancer survivors experience increasing BMI. Severity of obesity at diagnosis matters, patients with severe obesity (Class III) were most likely to experience weight gain.

## Highlights

• A third of endometrial cancer patients experience weight gain after diagnosis.
• Class III obesity is associated with increasing BMI after endometrial cancer diagnosis.
• Estimated median income is associated with BMI in endometrial cancer patients.

## 1 Introduction

Endometrial cancer is the most common gynecologic malignancy in the United States (Siegel et al., 2022). The most common subset; type I endometrial cancer, is directly related to obesity and metabolic syndrome (Bokhman, 1983, Creasman, 2002). Over 55% of women diagnosed with endometrial cancer are obese (Calle and Kaaks, 2004, Renehan et al., 2008). In 2022; it is estimated there will be 65, 950 new cases of endometrial cancer and 12,550 endometrial cancer deaths in the United States (Siegel et al., 2022); these numbers are expected to continue to rise due to increasing rates of obesity and metabolic syndrome.

Obesity has several mechanisms of action to drive endometrial proliferation and tumorigenesis (Onstad et al., 2016). Excess adiposity results unopposed estrogen through the peripheral conversion of androgens leading to abnormal endometrial proliferation, inflammation, and cancer (Creasman, 2002, Kaaks et al., 2002, Lépine et al., 2010). Adipose tissue is also rich in adipokines that regulate expression of sex hormone binding globin which influences systemic levels of estrogen (Onstad et al., 2016). Estrogen directly mediates endometrial proliferation through its effect on transcription factors (Lépine et al., 2010). Obesity related pro-inflammatory adipokines modulate insulin signaling and contribute to insulin resistance. Patients with Type 2 diabetes have increased expression of insulin and insulin-like growth factor 1 (IGF1) receptor, which has also been associated with endometrial hyperplasia (McCampbell et al., 2006). Increased expression of insulin and IGF1 promotes downstream signaling of PI3K/AKT and MAPK pathways that have been implicated in endometrial tumorigenesis (Onstad et al., 2016).

Fortunately, type 1 endometrial cancer is often detected early, and most patients are cured with primary therapy. There are over 1 million endometrial cancer survivors in the United States (Miller et al., 2019). The number one cause of mortality among women who survive endometrial cancer is premature death due to cardiovascular disease (Soisson, 2018, Ward et al., 2012, Felix et al., 2017, Sturgeon et al., 2019). Type 2 diabetes and metabolic syndrome are the greatest risk factors for cardiovascular disease (CVD) among endometrial cancer survivors (Aleksandrova et al., 2014, Alberti et al., 2005). Endometrial cancer survivors are often encouraged to mitigate their risk for metabolic syndrome through diet and exercise. To date, lifestyle intervention studies have shown conflicting results on weight loss for endometrial cancer survivors (McCarroll et al., 2014, Kitson et al., 2018, Haggerty et al., 2016, von Gruenigen, 2009, von Gruenigen et al., 2008, Koutoukidis et al., 2019, Zamorano et al., 2021). Prior to designing further interventions, we must identify who is at greatest risk for weight gain after endometrial cancer diagnosis. The primary objective of this study is to describe risk factors associated with increased body mass index (BMI) trajectory among endometrial cancer patients.

## 2 Methods

Institutional Review Board approval for the study was obtained through the University of Kansas Medical Center. Using previously described methods, “HERON” (Healthcare Enterprise Repository for Ontological Narration) (Waitman, 2011, Petersen et al., 2021) was used to identify patients with surgically treated endometrial cancer (using ICD-9 and ICD-10 codes) between 2010 and 2015. Patients with uterine sarcomas were excluded.

## 3 Measures

Clinical characteristics including age at diagnosis, race/ethnicity, stage at diagnosis, receipt of adjuvant therapy, weight and height were obtained. To evaluate weight changes over time, serial height (m) and weight (kg) measurements were abstracted for the following time points after diagnosis: baseline, 3 months, 6 months, 1 year, 1.5 years and 2 years. Body mass index (BMI) was calculated using the standard kg/m^2^ calculation. BMI classifications were as follows, underweight < 18.5 kg/m^2^, normal BMI (BMI 18.5–24.9 kg/m^2^), overweight (BMI 25–29.9 kg/m^2^) and obese (BMI > 30 kg/m^2^). Obesity was classified into 3 groups, Class I obesity (BMI 30 to 34.9 kg/m^2^), Class II obesity (BMI 35 to 39.9 kg/m^2^) and Class III obesity (BMI > 40 kg/m^2^), also referred to as severe obesity (Weir and Jan, 2022). Diagnoses of hypertension, diabetes, sleep apnea, depression and anxiety were also abstracted. As a measure of socioeconomic status, median income was estimated using the 2013 American Census Survey tables by matching on state, county, tract and block group (or zip code if the address is a P.O. Box).

## 4 Statistical analysis

BMI trajectories were estimated by latent class growth modeling using the PROC TRAJ procedure in SAS. The censored normal distribution was used. The number of distinct trajectories that best fit the data was three. These three groups were categorized into “increasing,” “decreasing,” and “stable” based on visual inspection of the slope.

Summary statistics were used to describe patient demographics and clinical characteristics. Chi-squared tests and ANOVA were used to assess differences in demographics and clinical characteristics between trajectory groups for categorical and continuous data, respectively. A subgroup analysis of patients with BMI ≥ 30 at baseline was conducted using similar methods. Statistical significance was set to a p-value < 0.05.

## 5 Results

A total of 695 patients who underwent hysterectomy for endometrial cancer were included in the study. Patient demographics are shown in Table 1. The average age at diagnosis was 62 years of age. Most patients were white and diagnosed with stage I disease (75%). Twenty percent of patients received adjuvant radiation and 15% received adjuvant chemotherapy. Only 0.6% (n = 4) of patients were underweight at diagnosis, 9.6% (n = 67) of patients had a normal BMI (BMI 18.5–24.9 kg/m^2^), 16% (n = 111) were overweight (BMI 25–29.9 kg/m^2^) and most patients 73.9% (n = 513) were obese (BMI > 30 kg/m^2^) at diagnosis. The average baseline BMI was 37 kg/m^2^. All patients had baseline BMI measurements (n = 695), 94% (n = 656) had 3-month BMI measurements, 72% (n = 497) had 6-month measurements, 64% (n = 446) had 1 year BMI measurements, 54% (n = 378) had 1.5-year BMI measurements and 53% (n = 366) had 2-year BMI measurements.

**Table 1:** Patient demographics and clinical characteristics.

Characteristic	Overall (n = 695)
Mean age (years) ± SD	62.2 ± 11.2
Race White Black Asian Am Indian/Pacific Isl Declined Other	588 (84.6%) 44 (6.3%) 2 (0.3%) 8 (1.2%) 3 (0.4%) 50 (7.2%)
Stage at diagnosis Stage IA Stage IB Stage II Stage IIIA Stage IIIB Stage IIIC1 Stage IIIC2 Stage IVA Stage IVB	459 (66%) 64 (9.2%) 82 (11.8%) 8 (1.2%) 4 (0.6%) 51 (7.3%) 11 (1.6%) 8 (1.2%) 8 (1.2%)
Histology Endometrioid Clear Cell Serous Mixed Cell Carcinosarcoma Mucinous Endometrial Stromal Undifferentiated	623 (89.6%) 2 (0.3%) 21 (3%) 28 (4%) 11 (1.6%) 1 (0.2%) 6 (0.9%) 3 (0.4%)
Adjuvant radiation therapy Yes No	139 (20%) 556 (80%)
Adjuvant chemotherapy Yes No	107 (15.4%) 588 (84.6%)
Baseline BMI (kg/m^2^) ± SD	37.0 ± 10.3
Baseline BMI category Underweight Normal Overweight Obese	4 (0.6%) 67 (9.6%%) 111 (16%) 513 (73.9%)
Estimated Median Income ($)	55,495

**Fig. 1:**
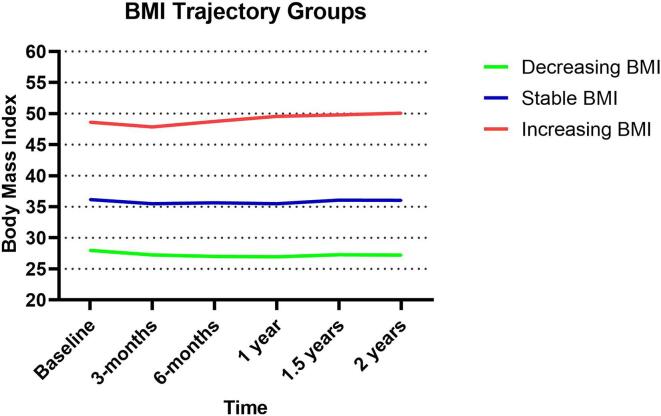
Three BMI trajectory groups.

Using trajectory modeling, we found three trajectory groups that best fit the data. The trajectory groups were described as 1. decreasing BMI, 2. stable BMI and 3. increasing BMI. (Fig. 1) The baseline BMI of patients in the trajectory groups were statistically different. The mean baseline BMI in patients in the increasing BMI trajectory group (group 3) was significantly higher than those in the decreasing BMI trajectory group (group 1), 50 kg/m2 vs 26.8 kg/m2, p < 0.0001. All patients with a normal baseline BMI were in the decreasing BMI trajectory group. Similarly, 97.3% of patients with an overweight baseline BMI were also in the decreasing BMI trajectory group. In contrast, all patients in the increasing BMI trajectory group had a baseline obesity.

The average age of women in the decreasing BMI trajectory group was significantly higher than women in the stable or increasing BMI trajectory group, 65.1 years vs 62.4 years vs 58.5 years respectively, p < 0.0001. Similarly, women aged 70 years and older were more likely to be in the decreasing BMI trajectory group than the stable or increasing BMI groups, 37.8% vs 22.3% vs 9.8% respectively, p < 0.0001. After controlling for age at diagnosis, these three BMI trajectory groups still best fit the data.

Patients in the increasing BMI trajectory group were more likely to have Stage IA at diagnosis and endometrioid histology compared to the stable or decreasing BMI trajectory groups (Table 2). Patients in the decreasing BMI trajectory group were more likely to have non-endometrioid histology, with 15% of patients presenting with type II cancers. Patients in the decreasing BMI group were also more likely to receive adjuvant radiation and chemotherapy. The decreasing BMI trajectory group had the lowest percentage of patients (55%) with Stage IA at diagnosis. After controlling for stage at diagnosis, these three BMI trajectory groups still best fit the data.

**Table 2:** Patient characteristics by trajectory grouping.

Characteristic	Overall N = 695	Decreasing Trajectory N = 233	Stable Trajectory N = 269	Increasing Trajectory N = 193	p-value
Age at diagnosis ± SD (mean years)	62.2 ± 11.2	65.1 ± 12.5	62.4 ± 10.7	58.5 ± 9.2	<0.0001
Age ≥ 70 Age < 70	167 (24%) 528 (76%)	88 (37.8%) 145 (62.2%)	60 (22.3%) 209 (77.7%)	19 (9.8%) 174 (90.2%)	<0.0001
Race White Black Asian Am Indian/Pacific Isl Declined Other	588 (84.6%) 44 (6.3%) 2 (0.3%) 8 (1.2%) 3 (0.4%) 50 (7.2%)	196 (84.1%) 12 (5.2%) 2 (0.9%) 3 (1.3%) 0 (0%) 20 (8.6%)	224 (83.3%) 20 (7.4%) 0 (0%) 2 (0.7%) 1 (0.4%) 22 (8.2%)	168 (87.1%) 12 (6.2%) 0 (0%) 3 (1.6%) 2 (1%) 8 (4.2%)	0.2822
Stage at diagnosis Stage IA Stage IB Stage II Stage IIIA Stage IIIB Stage IIIC1 Stage IIIC2 Stage IVA Stage IVB	459 (66%) 64 (9.2%) 82 (11.8%) 8 (1.2%) 4 (0.6%) 51 (7.3%) 11 (1.6%) 8 (1.2%) 8 (1.2%)	129 (55.4%) 27 (11.6%) 37 (15.9%) 5 (2.2%) 3 (1.3%) 21 (9%) 4 (1.7%) 4 (1.7%) 3 (1.3%)	182 (67.7%) 23 (8.6%) 26 (9.7%) 2 (0.7%) 1 (0.4%) 24 (8.9%) 4 (1.5%) 3 (1.1%) 4 (1.5%)	148 (76.7%) 14 (7.3%) 19 (9.8%) 1 (0.5%) 0 (0%) 6 (3.1%) 3 (1.6%) 1 (0.5%) 1 (0.5%)	0.0179
Histology Endometrioid Clear Cell Serous Mixed Cell Carcinosarcoma Mucinous Undifferentiated	623 (89.6%) 2 (0.3%) 21 (3%) 28 (4%) 11 (1.6%) 1 (0.1%) 9 (1.3%)	198 (85%) 1 (0.4%) 13 (5.6%) 8 (3.4%) 7 (3%) 0 (0%) 6 (2.6%)	242 (90%) 1 (0.4%) 8 (3%) 10 (3.7%) 4 (1.5%) 1 (0.4%) 3 (1.2%)	183 (94.8%) 0 (0%) 0 (0%) 10 (5.2%) 0 (0%) 0 (0%) 0 (0%)	0.0086
Adj.Radiation Therapy Yes No	139 (20%) 556 (80%)	60 (25.8%) 173 (74.3%)	54 (20%) 215 (80%)	25 (13%) 168 (87%)	0.0045
Adj. chemotherapy Yes No	107 (15.4%) 588 (84.6%)	47 (20.2%) 186 (79.8%)	45 (16.7%) 224 (83.3%)	15 (7.8%) 178 (92.2%)	0.0015
BMI ± SD (kg/m^2^)	37.0 ± 10.3	26.8 ± 3.7	36.4 ± 3.3	50 ± 7.5	<0.0001
BMI category Underweight Normal Overweight Obese	4 (0.6%) 67 (9.6%) 111 (16%) 513 (73.8%)	4 (1.7%) 67 (28.8%) 108 (46.4%) 54 (23.2%)	0 (0%) 0 (0%) 3 (1.1%) 266 (98.9%)	0 (0%) 0 (0%) 0 (0%) 193 (100%)	<0.0001
Hypertension Yes No	347 (49.9%) 348 (50.1%)	97 (41.6%) 136 (58.4%)	137 (50.9%) 132 (49.1%)	113 (58.6%) 80 (41.4%)	0.0022
Diabetes Yes No	163 (23.5%) 532 (76.6%)	31 (13.3%) 202 (86.7%)	72 (26.8%) 197 (73.2%)	60 (31.1%) 133 (68.9%)	<0.0001
Sleep Apnea Yes No	46 (6.6%) 649 (93.4%)	4 (1.7%) 229 (98.3%)	13 (4.8%) 256 (95.2%)	29 (15%) 164 (85%)	<0.0001
Depression Yes No	71 (10.2%) 624 (89.8%)	22 (9.4%) 211 (90.6%)	24 (8.9%) 245 (91.1%)	25 (13%) 168 (87%)	0.3296
Anxiety Yes No	62 (8.9%) 633 (91.1%)	16 (6.9%) 217 (93.1%)	27 (10%) 242 (90%)	19 (9.8%) 174 (90.2%)	0.4015
Median Income ($)	55,495	60,192	55,057	50,474	0.0010

Patients with hypertension were more likely to experience increasing BMI over time rather than stable or decreasing BMI, p = 0.0022. Patients with diabetes were also more likely to experience increasing BMI over time than the decreasing or stable BMI trend, p < 0.0001. Similarly, patients with sleep apnea were also more likely to be in the increasing BMI trajectory group than the other two groups, p < 0.0001. Depression and anxiety were not statistically associated with trajectory groupings. **(**Table 2**)** In terms of socioeconomic status, patients who experienced increasing BMI over time were more likely to have lower median income ($50,474), than those who had stable BMI ($55,057) and those who had decreasing BMI ($60,192) over time, p = 0.0010.

Subgroup analyses of women who were obese at baseline (n = 513) demonstrated that patients who were in the decreasing BMI trajectory group and obese at baseline (n = 54) were older than those in the stable and increasing trajectory groups. While all patients in this subgroup analysis were obese, the severity of obesity differed, 98% of patients in the decreasing trajectory group had Class I obesity whereas 99% of patients in the increasing BMI trajectory had Class III obesity. There was no difference in stage at diagnosis, histology, or receipt of adjuvant radiation therapy among BMI trajectory groups of patients who were obese at baseline. There was a higher proportion of patients who received chemotherapy in the stable BMI trajectory group than the decreasing or increasing BMI groups, 16.2% vs 14.8%, vs 7.8% respectively, p = 0.0269, (Table 3). There was no difference in rates of diabetes, hypertension, anxiety or depression among patients who were obese at baseline. The presence of sleep apnea was associated with the increasing BMI trajectory. Although not statistically significant, there was a trend of higher estimated median income of obese patients in the decreasing BMI trajectory group than those obese patients with stable and increasing BMI (p = 0.0790). (Table 3**)**

**Table 3:** Subgroup analysis of patients with BMI ≥ 30 kg/m^2^ at baseline.

Characteristic	Overall N = 513	Decreasing Trajectory N = 54	Stable Trajectory N = 266	Increasing Trajectory N = 193	p-value
Age at diagnosis ± SD (mean years)	61.4 ± 10.3	65.9 ± 10.9	62.6 ± 10.4	58.5 ± 9.2	<0.0001
Age ≥ 70 Age < 70	100 (19.5%) 413 (80.5%)	22 (40.7%) 32 (59.3%)	59 (22.2%) 207 (77.8%)	19 (9.9%) 174 (90.1%)	<0.0001
Race White Black Asian Am Indian/Pacific Isl Declined Other	435 (84.8%) 36 (7%) 0 (0%) 6 (1.2%) 3 (0.6%) 33 (6.4%)	46 (85.2%) 4 (7.4%) 0 (0%) 1 (1.8%) 0 (0%) 3 (5.6%)	221 (83.1%) 20 (7.5%) 0 (0%) 2 (0.7%) 1 (0.4%) 22 (8.3%)	168 (87%) 12 (6.2%) 0 (0%) 3 (1.6%) 2 (1%) 8 (4.2%)	0.6957
Stage at diagnosis Stage IA Stage IB Stage II Stage IIIA Stage IIIB Stage IIIC1 Stage IIIC2 Stage IVA Stage IVB	358 (69.8%) 41 (8%) 54 (10.5%) 4 (0.8%) 2 (0.4%) 33 (6.4%) 9 (1.8%) 5 (1%) 7 (1.4%)	29 (53.7%) 4 (7.4%) 9 (16.7%) 1 (1.8%) 1 (1.8%) 5 (9.3%) 2 (3.7%) 1 (1.8%) 2 (3.7%)	181 (68.1%) 23 (8.7%) 26 (9.8%) 2 (0.7%) 1 (0.4%) 22 (8.3%) 4 (1.5%) 3 (1.1%) 4 (1.5%	148 (76.7%) 14 (7.3%) 19 (9.8%) 1 (0.5%) 0 (0%) 6 (3.1%) 3 (1.5%) 1 (0.5%) 1 (0.5%)	0.1624
Histology Endometrioid Clear Cell Serous Mixed Cell Carcinosarcoma Mucinous Undifferentiated	472 (92%) 1 (0.2%) 9 (1.8%) 22 (4.3%) 6 (1.2%) 1 (0.2%) 2 (0.4%)	47 (87%) 0 (0%) 1 (1.8%) 4 (7.4%) 2 (3.7%) 0 (0%) 0 (0%)	242 (91%) 1 (0.4%) 8 (3%) 8 (3%) 4 (1.5%) 1 (0.4%) 2 (0.8%)	183 (94.8%) 0 (0%) 0 (0%) 10 (5.2%) 0 (0%) 0 (0%) 0 (0%)	0.2112
Adj.Radiation Therapy Yes No	88 (17.1%) 425 (82.9%)	10 (18.5%) 44 (81.5%)	53 (19.9% 213 (80.1%)	25 (13%) 168 (87%)	0.1420
Adj. chemotherapy Yes No	66 (12.9%) 447 (87.1%)	8 (14.8%) 46 (85.2%)	43 (16.2%) 223 (83.8%)	15 (7.8%) 178 (92.2%)	0.0269
BMI ± SD (kg/m^2^)	41.1 ± 8.8	31.2 ± 1.5	36.5 ± 7.8	50 ± 7.5	<0.0001
Obesity Class I (BMI 30 to 34.9 kg/m^2^) II (BMI 35 to 39.9 kg/m^2^) III (BMI > 40 kg/m^2^)	149 (29%) 128 (25%) 236 (46%)	53 (98.2%) 0 (0%) 1 (1.8%)	95 (35.7%) 127 (47.7%) 44 (16.5%)	1 (0.5%) 1 (0.5%) 191 (99%)	<0.0001
Hypertension Yes No	275 (53.6%) 238 (46.4%)	26 (48.1%) 28 (51.9%)	136 (51.1%) 130 (48.9%)	113 (58.6%) 80 (41.4%)	0.2019
Diabetes Yes No	145 (28.3%) 368 (71.7%)	13 (24.1%) 41 (75.9%)	72 (27.1%) 194 (72.9%)	60 (31.1%) 133 (68.9%)	0.4930
Sleep Apnea Yes No	44 (8.6%) 469 (91.4%	2 (3.7%) 52 (96.3%)	13 (4.9%) 253 (95.1%)	29 (15%) 164 (85%)	0.0003
Depression Yes No	56 (10.9%) 457 (89.1%)	8 (14.8%) 46 (85.2%)	23 (8.7%) 243 (91.3%)	25 (13%) 168 (87%)	0.2147
Anxiety Yes No	49 (9.6%) 464 (90.4%)	3 (5.6%) 51 (94.4%)	27 (10.2%) 239 (89.8%)	19 (9.8%) 174 (90.2%)	0.5690
Median Income ($)	53,643	56,647	55,340	50,474	0.0790

## 6 Discussion

This is a retrospective study that has identified three BMI trends among endometrial cancer patients in the two years following diagnosis. Of 695 endometrial cancer patients, 33.5% experienced decreasing BMI, 38.7% experienced stable BMI and 27.8% experienced increased BMI. These findings are consistent with a previous study which determined 30.2% of endometrial cancer patients experienced increasing obesity in the two years after diagnosis (Matsuo et al., 2016). However, characteristics of patients most at risk for weight gain were not identified in that analysis. In our study, younger age at diagnosis, lower estimated median income, and higher BMI at diagnosis were identified as risk factors associated with increased BMI trajectory. Those with diabetes, sleep apnea and hypertension were also more likely to have an increasing BMI trend.

We have shown that patients in the decreasing BMI trajectory group had higher rates of non-endometrioid histology and lower baseline BMI at diagnosis. This is consistent with prior studies that show type II endometrial cancers are less likely to be associated with obesity (Setiawan, 2013). Patients in the decreasing BMI trajectory group were also more likely to receive adjuvant radiation and chemotherapy, likely due to higher rates of type II cancers. Receipt of adjuvant treatment may present a source of confounding as patients often experience weight loss with chemotherapy and radiation. However, in our subgroup analysis of only obese patients, there was no difference in stage at diagnosis, histology, or receipt of radiation therapy between BMI trajectory groups. In fact, the stable BMI trajectory group had a higher proportion of patients who received adjuvant chemotherapy than the increasing or decreasing BMI trajectory groups. Thus, we do not believe that receipt of adjuvant therapy serves as a major source of confounding in this study.

Over 70% of patients in this study were obese at diagnosis, signaling the burden of obesity in the endometrial cancer population. Patients with Class III obesity at diagnosis were at an increased risk for weight gain. The Society of Gynecologic Oncology recommends that cancer survivors achieve a healthy lifestyle including maintaining a healthy weight, eating a balanced diet and incorporating regular exercise (Oncology, 2018). Weight management should be discussed with patients at diagnosis, during treatment and as a part of survivorship. Unfortunately, weight loss interventions in endometrial cancer survivors have not been widely successful and currently there is no best practice guideline for decreasing rates of obesity among survivors (Kitson et al., 2018).

Although obesity is a known risk factor for endometrial cancer, obese endometrial cancer survivors often underestimate their obesity and lack an understanding of the link between obesity and endometrial cancer (Haggerty et al., 2017). In fact, there is a general lack of awareness of the link between obesity and endometrial cancer. Patients obtaining routine gynecologic care are often unaware of the relationship between obesity and endometrial cancer regardless of their education level (Washington et al., 2020). The American Society of Clinical Oncology recommends that public awareness of the link between obesity and cancer be emphasized to help reduce obesity and modify cancer risk (Ligibel et al., 2017). The Women’s Preventive Services Initiative has recently recommended weight management counseling to prevent obesity in midlife women with normal or overweight BMI (Chelmow, 2022). To date, there has not been a national campaign to raise awareness of risk factors for endometrial cancer although there is increasing discussion in the lay media about the rising incidence of endometrial cancer in the United States.

We believe that endometrial cancer survivorship may be a teachable moment to discuss the impact of obesity on diagnosis and how lifestyle changes can affect health outcomes. However, we recognize that socioeconomic status limits endometrial cancer patients’ ability access obesity-related resources (Ross et al., 2019, Wilson, 2021). In this study, patients who experienced weight gain after diagnosis were also more likely to have a lower estimated median income by an average of $10,000 annually. Socioeconomic status is associated with obesity, through effects on access to fresh foods, green space and accessibility to safe spaces for physical activity as well as a potential limited ability to prioritize self-care and mitigate stress levels due to financial and caretaking constraints. Endometrial cancer survivors living in the lowest socioeconomic status conditions in Chicago had higher rates of obesity, obesity related comorbidities and the poorest access to recommended obesity related resources (Ross et al., 2019).

We also recognize the disproportionate burden of obesity on communities of color. While the impact of race/ethnicity on BMI trajectory was not investigated in this majority white cohort, racial disparities exist in the incidence of obesity in the United States. >50% of non-Hispanic Black women are obese as compared to 17% of non-Hispanic white women (Hales, 2020). In Chicago, Black endometrial cancer survivors live in the lowest socioeconomic status conditions and have the poorest access to exercise, weight management and diet resources (Ross et al., 2019).

To our knowledge, this is the first study to identify factors associated with increased BMI trajectory in endometrial cancer patients. However, this is a retrospective study with inherent potential for errors in reporting and unknown potential confounders that may prevent generalization. For instance, we could not determine if weight loss was intentional or if patients participated in lifestyle modification programs. Additionally, there was loss of data over BMI time points with 53% of patients having a BMI measurement at 2 years. However, the SAS Trajectory Procedure (Proc Traj) is designed to identify subgroups within a population, estimate the pattern of change over time, and assume that every subject in the group follows the same trajectory, therefore, some missing data does not adversely affect the outcome (Arrandale, 2006, Nagin and Odgers, 2010, Jones et al., 2001). Although Proc Traj is a powerful tool, it cannot provide individual level information on the pattern of change over time, thus, we are unable to estimate individual weight gain or loss (Arrandale, 2006, Nagin and Odgers, 2010). We did not examine BMI trajectories for patients beyond the first 2 years of survivorship, so we cannot determine risk factors for increased BMI into the late survivorship period. Another potential limitation is the use of American Census Survey data to estimate median income based on a patient’s residence, race and household size. This may over or underestimate the median income for patients in the study but is a practical way to obtain income data.

In conclusion, we have shown that a third of endometrial cancer patients experience increasing BMI after diagnosis. These patients have a younger age, lower estimated median income, and a higher BMI at diagnosis. We believe that multidisciplinary interventions to reduce rates of obesity among endometrial cancer survivors should be prioritized and delivered to the highest risk groups. While outside the scope of this study, we also acknowledge the impact of structural racism on the prevalence of obesity in communities of color and on the increased incidence of endometrial cancer among Black women. We recommend that future studies evaluate BMI trends in the late survivorship period beyond two years and that weight loss interventions prioritize the highest risk and most vulnerable groups of women.

### CRediT authorship contribution statement

**Shariska Petersen Harrington:** Conceptualization, Writing – original draft, Writing – review & editing, Funding acquisition. **Julia Balmaceda:** Writing – review & editing. **Lori Spoozak:** Writing – review & editing. **Andrea Jewell:** Writing – review & editing. **Sharon Fitzgerald-Wolff:** Writing – review & editing, Supervision.

## Declaration of Competing Interest

The authors declare the following financial interests/personal relationships which may be considered as potential competing interests: Shariska Petersen Harrington: This work was supported by a CTSA grant from NCATS awarded to the University of Kansas (# TL1TR002368). This work was a result of TREC Training Workshop R25CA203650 (PI: Melinda Irwin). HERON is funded by CTSA Award #UL1TR002366.
